# Evaluating the Disease-Related Experiences of TikTok Users With Lupus Erythematosus: Qualitative and Content Analysis

**DOI:** 10.2196/51211

**Published:** 2024-04-17

**Authors:** Lindsey J Wanberg, David R Pearson

**Affiliations:** 1 University of Minnesota Medical School Minneapolis, MN United States; 2 Department of Dermatology University of Minnesota Minneapolis, MN United States

**Keywords:** lupus, TikTok, autoimmune disease, qualitative research, quality of life

## Abstract

**Background:**

Lupus erythematosus (LE) is an autoimmune condition that is associated with significant detriments to quality of life and daily functioning. TikTok, a popular social networking platform for sharing short videos, provides a unique opportunity to understand experiences with LE within a nonclinical sample, a population that is understudied in LE research. This is the first qualitative study that explores LE experiences using the TikTok platform.

**Objective:**

This study aims to evaluate the disease-related experiences of TikTok users with LE using qualitative and content analysis.

**Methods:**

TikTok videos were included if the hashtags included #lupus, were downloadable, were in English, and involved the personal experience of an individual with LE. A codebook was developed using a standardized inductive approach of iterative coding until saturation was reached. NVivo (Lumivero), a qualitative analysis software platform, was used to code videos and perform content analysis. Inductive thematic analysis was used to derive themes from the data.

**Results:**

A total of 153 TikTok videos met the inclusion criteria. The most common codes were *experiences with symptoms* (106/153, 69.3%), *mucocutaneous symptoms* (61/153, 39.9%), and *experiences with treatment* (59/153, 38.6%). *Experiences with symptoms* and *mucocutaneous symptoms* had the greatest cumulative views (25,381,074 and 14,879,109 views, respectively). Five thematic conclusions were derived from the data: (1) mucocutaneous symptoms had profound effects on the mental health and body image of TikTok users with LE; (2) TikTok users’ negative experiences with health care workers were often derived from diagnostic delays and perceptions of “medical gaslighting”; (3) TikTok users tended to portray pharmacologic and nonpharmacologic interventions, such as diet and naturopathic remedies, positively, whereas pharmacologic treatments were portrayed negatively or referred to as “chemotherapy”; (4) LE symptoms, particularly musculoskeletal symptoms and fatigue, interfered with users’ daily functioning; and (5) although TikTok users frequently had strong support systems, feelings of isolation were often attributed to battling an “invisible illness.”

**Conclusions:**

This study demonstrates that social media can provide important, clinically relevant information for health practitioners caring for patients with chronic conditions such as LE. As mucocutaneous symptoms were the predominant drivers of distress in our sample, the treatment of hair loss and rash is vital in this population. However, pharmacologic therapies were often depicted negatively, reinforcing the significance of discussions on the safety and effectiveness of these treatments. In addition, while TikTok users demonstrated robust support systems, feelings of having an “invisible illness” and “medical gaslighting” dominated negative interactions with others. This underscores the importance of providing validation in clinical interactions.

## Introduction

### Background

The term lupus erythematosus (LE) encompasses a group of autoimmune disorders that may have multiorgan involvement, as in systemic LE (SLE), which affects >3.4 million individuals worldwide, or have primarily cutaneous manifestations, as in discoid LE [1,2]. Patients with LE frequently experience detriments to quality of life and daily functioning [3,4]. Because of this, a number of qualitative studies were conducted over the last decade that attempted to better understand patient experiences with LE [5]. However, the large majority of these studies recruited participants from clinical settings; thus, clear gaps persist in understanding how experiences with LE can be improved in individuals outside the health care system [3,5].

Social media is underused in the qualitative research of individuals with LE. To our knowledge, only 2 thematic analyses have been conducted using LE-related content on social media forums, including an analysis of comments on an LE Facebook group and an analysis of LE-related Twitter (since rebranded as X) posts [6,7]. Qualitative research using social media is important because it captures individuals who are understudied in typical qualitative research because social media users represent a nonclinical sample and thus may have varied experiences with, and accessibility to, health care [8]. In addition, with estimates suggesting that 40% to 45% of individuals use social media to make medical decisions, it is clinically prudent to determine how diseases such as LE are being portrayed to patients seeking information about their condition [9-11].

TikTok (ByteDance) is an extremely popular social media platform, with >1 billion monthly users [11]. TikTok users post short—often <1 minute—videos and can add filters, music, and captions to their content within the app. TikTok videos provide an untapped, novel, and abundant source of patient experiences to examine; nevertheless, they have yet to be used in thematic analysis within the fields of dermatology or rheumatology. In fact, #lupus has 1.3 billion cumulative views on TikTok alone, indicating the popularity and prevalence of the topic on the app [12]. Furthermore, because TikTok is the fastest growing social media platform worldwide, it is imperative to study it because it is a rapidly expanding source of health information for patients seeking knowledge on the web [9,11].

### Objectives

In this study, we used content analysis and thematic analysis to examine TikTok videos involving personal experiences with LE. By doing so, we hope to gain a better understanding of the disease-related experiences of TikTok users who have LE.

## Methods

### Data Collection

A new TikTok account was created for data collection to avoid TikTok algorithms that prioritize videos based on prior user activity [13,14]. The account was used to search for #lupus on the TikTok app on August 21, 2022; the app then displayed the most popular videos tagged with #lupus [13]. The links, captions, usernames, likes, comments, views, and shares were extracted from each video identified through this search. Videos were then evaluated for inclusion and exclusion criteria. Videos were included if they were downloadable, were in English, and involved the personal opinions or experiences of an individual with LE. They were excluded if they did not relate to LE or were primarily about a condition other than LE.

### Codebook Development

A codebook was developed using a standardized inductive approach of iterative coding [13,15]. Study team members coded sets of 20 TikTok videos determined through random selection. After independently coding each set, the study team members met to reach consensus on the codes used for each video. Next, they collaboratively assigned labels, definitions, exclusions, and examples to new codes for the purpose of developing a preliminary codebook [15]. This process was repeated until saturation was reached, that is, no new major codes were created or adjusted, and the codebook could be finalized (refer to Multimedia Appendix 1 for the finalized codebook) [15].

### Data Analysis

NVivo 2020 (Lumivero), a qualitative analysis software platform, was used for coding and analysis. Videos were imported into the NVivo software and transcribed. Line-by-line coding of each video’s transcript and caption was accomplished within NVivo using the finalized codebook. For the quantitative content analysis aspect of this study, the prevalence and frequency of overlap of individual codes was obtained through NVivo. For the top codes, median views and median views per day were calculated. For the qualitative analysis aspect of this study, the study team members met to discuss the most predominant and rich codes, and they used inductive thematic analysis to derive major themes from the data [16].

### Ethical Considerations

This research was determined by the University of Minnesota Institutional Review Board in July 2022 as not constituting human subjects research. Included videos had to be downloadable because this was seen as an indication that the user intended their content to be used and shared by others [13]. All identifying data, including usernames, were removed from the data before dissemination. The study team consulted with the University of Minnesota Medical School Office of Diversity, Equity, & Inclusion and elected not to document user demographics from individual videos because doing so would involve the assumption of identity through appearance on video, and objective demographic information of individual TikTok users is not publicly available.

## Results

### Overview

A total of 398 TikTok videos were identified through the #lupus search and underwent inclusion and exclusion criteria, after which 153 (38.4%) videos posted between December 19, 2019, and August 21, 2022, were included for analysis. Cumulatively, the videos had 29,446,765 views, with a median of 37,200 (IQR 146,936; range 163-2,300,000) views per video. A total of 76 distinct TikTok users were represented in the sample. Users were primarily female presenting. Of the 76 users, 3 (4%) contributed 35.9% (55/153) of the videos; the top user contributed 15.7% (24/153) of the videos, followed by a user with 11.8% (18/153) and a user with 8.5% (13/153). The most common codes were *experiences with symptoms* (107/153, 69.9%), *mucocutaneous symptoms* (62/153, 40.5%), and *experiences with treatment* (58/153, 37.9%). *Experiences with symptoms* and *mucocutaneous symptoms* had the greatest number of cumulative views (24,426,874 and 14,082,409 views, respectively; Table 1).

Five major thematic conclusions were derived from the data and are explored in the subsections that follow. Sample quotes for each theme can be found in Textbox 1.

**Table 1:** Top codes and their popularity metrics.

Codes	Videos coded (n=153), n (%)	Cumulative views	Views, median (IQR; range)	Views/d, median (IQR; range)
Experiences with symptoms	107 (69.9)	25,381,074	37,300 (201,100; 234-2,300,000)	582 (3732.5; 2-250,000)
Mucocutaneous symptoms	62 (40.5)	14,879,109	54,700 (280,700; 474-2,300,000)	699 (4507.3; 14-188,889)
Treatment experience	58 (37.9)	11,744,223	34,950 (170,334; 163-2,300,000)	1042 (6152.9; 1-188,889)
Health care experience	45 (29.4)	8,546,113	32,000 (123,322.5; 163-2,300,000)	582 (2315.8; 1-127,778)
Constitutional symptoms	43 (28.1)	9,993,593	31,100 (147,063; 234-2,300,000)	509 (4037.8; 3-250,000)
Mental health	42 (27.5)	7,703,071	32,150 (133,700; 234-2,300,000)	1748 (3391.5; 2-127,778)
Fatigue	41 (26.8)	9,835,548	31,100 (158,033; 234-2,300,000)	691 (4269.1; 3-250,000)
Hair loss	40 (26.1)	10,240,943	75,550 (311,900; 474-1,700,000)	733 (4526.5; 16-188,889)
Rash	39 (25.5)	8,767,466	43,800 (283,300; 474-1,700,000)	691 (4564.0; 14-188,889)
Humor	35 (22.9)	4,548,187	33,800 (57,683; 474-1,500,000)	545 (1263.6; 16-250,000)
Musculoskeletal symptoms	35 (22.9)	5,654,750	23,100 (83,400; 338-2,100,000)	165 (3627.2; 2-51,438)

**Summary of major themes with sample quotes, which have all been taken directly from users’ spoken words, written captions, or written subtitles.**

**Themes and TikTok user quotes**

- Mucocutaneous symptoms had profound effects on the mental health and body image of TikTok users with lupus erythematosus.
  - “In 2019, I had a full head of hair. Life can change in a blink of an eye. I was diagnosed with lupus in 2020. My hair was dropping like crazy. There was nothing I could do...[it was] painful and depressing. My scalp was filled with so many scabs. I couldn’t even touch my head. [I] kept saying to myself ‘this can’t be it.’” [User 67]
  - “I had to cut my hair because of lupus (
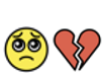
). I’ve been growing it for 7 and 1/2 years (
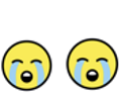
). My hair started thinning out at the top because of the lupus (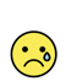). So I knew soon I would have to cut it...I just found out I had lupus 4 months ago (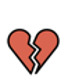). But at the end of the day (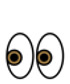), my health is more important than my hair and the only way I could get it to grow back right is if I cut it (
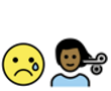
). This really broke my heart (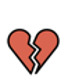) but I’m still handsome [17] (
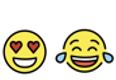
)”. [User 18]
- TikTok users’ negative experiences with health care workers were often derived from diagnostic delays and perceptions of “medical gaslighting.”
  - “Things doctors told me before I got diagnosed with lupus. Take some vitamins. Go outside more. You’re just stressed, take these antidepressants. It’s growing pains (I was 18). Have you tried yoga?” [User 33]
  - “How doctors would gaslight me until I was finally diagnosed:

[User portraying self] My body is aching so bad. I have really swollen lymph nodes and am losing weight fast.

[User portraying physician] Swollen lymph nodes are common. Maybe you’re losing weight because you’re depressed.

[User portraying self] I am so dizzy and have no energy. I am bruising everywhere and am always sick. Please, listen to me. I’m not making this up. Something is not right.

[User portraying physician] Are you exercising enough? You’re too young to have something serious going on.

[User providing commentary] This was my experience for almost 3 years. Begging doctors to take me seriously. It traumatized me. You are your biggest advocate.” [User 34]

- TikTok users tended to portray nonpharmacologic interventions, such as diet and naturopathic remedies, positively, whereas pharmacologic treatments were more commonly portrayed negatively or referred to as “chemotherapy.”
  - “I did my part by reporting new symptoms to my rheumatologist and neurologist, which of course was downplayed. Five months later, I lost my mobility and couldn’t do much for myself. I thought that I’d never bounce back. I researched natural practices/herbs and started a personal healing journey. I’ve regained my mobility and much more within a few months.” [User 28]
  - “Nightshade vegetables will cause you extreme pain in the long run if you’re someone who is dealing with autoimmune disease. I have lupus, but I put lupus in remission as quick as it came out of remission. So, my suggestion to you, unbeknownst to most people, stop eating nightshade vegetables. If you’ve got a garden, stop growing them. You’d be surprised, you could cure lupus immediately, just stop it.” [User 21]
  - “Today is one full week on chemo. I don’t want to keep doing this. [User 34]
  - “Sometimes you need things like chemotherapy...which sounds crazy, but [lupus] is that serious.” [User 8]
  - [User portraying physician] Doctor: I’m sorry, but in order to control your flare we need to start steroids.

[User portraying self] My face: [user uses a special effect that causes their face to blow up to 3 times its size and resemble the front of a train. Audio of a train horn plays in the background.]

[Caption] All aboard the moon face express (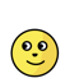) [17]”. [User 7]

- Lupus erythematosus symptoms, particularly musculoskeletal symptoms and fatigue, interfered with users’ daily functioning.
  - “I was diagnosed with lupus almost four years ago. And lupus took a lot from me...lupus stole my social life. It took my freedom, it destroys my energy, it took my job. Do you know what it’s like to be told you can’t work? I’ve had to adjust to a new normal. This sucks but I can’t let it win. That’s why I can assure you I will not give up. I’ve come so far, I can’t let it win.” [User 16]
  - “A lot of days, my body hurts so badly that I don’t possibly know how to get out of bed or survive for the next few hours.” [User 70]
- Although TikTok users frequently had strong support systems, feelings of isolation or misunderstanding were often attributed to battling an “invisible illness.”
  - “A true story about finding an *amazing* friend that actually gets it. She...is 100% understanding when I have to flake last minute because I don’t feel well. No guilt. A rare gem indeed.” [User 43]
  - “[Footage plays of user getting their nails painted by their husband] I have lupus. My husband helps me through the ups and downs of this...He doesn’t care about doing things for me. Swollen ankles, messed up toes, and constant pain. This is where we’re at in life. 19 years together.” [User 71]
  - “*Getting through life with lupus* Professors: Not understanding what I need. Most friends: Thinking I’m fine and insulting me unintentionally. Family: Making me feel like I’m doing it alone.” [User 12]

### Theme 1: Mucocutaneous Symptoms Had Profound Effects on the Mental Health and Body Image of TikTok Users With LE

Mucocutaneous symptoms, defined as hair loss, rash, photosensitivity, ulcers, itch, or sicca symptoms, were the most frequently mentioned symptoms of LE in our sample; 40.5% (62/153) of the videos referred to a mucocutaneous manifestation of LE. Hair loss and rash were the most common and were each coded in approximately one-quarter of the videos (40/153, 26.1% and 39/153, 22.5%, respectively).

Mucocutaneous symptoms were highly distressing to users, affecting both body image and mental well-being. Of the 153 videos, 24 (15.7%) involved a user exhibiting negative body image, of which 79% (n=19) were related to hair loss, 46% (n=11) were related to rash, and 92% (n=22) were related to either hair loss or rash. Furthermore, *hair loss* was the second most common code to overlap with *mental health*; nearly one-fifth (29/153, 19%) of the *mental health* codes directly overlapped with *hair loss* codes.

Distress and body image concerns were apparent within TikTok videos that mentioned mucocutaneous symptoms. Users felt that their hair and skin changes led to a loss of identity:

Going through losing all my hair was really hard for me. Like, I didn’t realize how much of my self-worth I attached to my hair...I felt like I was losing a piece of myself...I felt so sad all the time. And it was so hard for me to just go outside because I felt so, you know, insecure.

User 27

Although hair loss and rash had significant impacts on users’ well-being and body image, these effects appeared to be mitigated by cosmetic measures and pharmacologic and nonpharmacologic treatments. Of the 24 videos, 13 (54%) involved users cosmetically modifying their hair with sew-ins, wigs, hair dye, hairstyles, haircuts, or scarves to hide hair loss or improve confidence. A user stated as follows:

In 2016 I lost my hair...this was before the shaved head hype. Back then my hair was my identity...I knew I needed to cover it up because I had to go into work, so I did a few scarf tutorials and ended up like this. I mean, I think it looks pretty dope.

User 16

Skin-directed treatments also helped users. A user showed old footage of large clumps of their hair that had fallen out in the sink. The user recalled how they felt at that time:

Super stressed. I’m going to be bald. My hair won’t go back to normal.

User 68

They then showed footage of dozens of boxes of prednisone and their scalp with hair growth, stating as follows:

OMG! It might be working...Now I can do the hair styles I want.

User 68

### Theme 2: TikTok Users’ Negative Experiences With Health Care Workers Were Often Derived From Diagnostic Delays and Perceptions of “Medical Gaslighting”

Of the 153 videos, 45 (29.4%) involved a health care experience, of which 58% (n=26) depicted negative experiences, whereas 18% (n=8) depicted positive experiences. Of the 25 negative health care experiences, 22 (88%) could be attributed to an experience with a health care worker. Primarily, TikTok users expressed frustration due to diagnostic delays and “medical gaslighting,” which made up 64% (14/22) and 36% (8/22) of the negative interactions with health care workers, respectively (Textbox 2). Diagnostic delays described by users spanned from “months” to “years,” with 2 (14%) of the 14 users describing delays of ≥10 years.

In our sample, *diagnostic delays* and *medical gaslighting* frequently overlapped; 7 (4.6%) of all videos (n=153) described scenarios where users felt that their symptoms were belittled by medical professionals, leading to delayed diagnoses of LE. A TikTok user stated as follows:

[D]octors would gaslight me until I was finally diagnosed...this was my experience for almost 3 years. Begging doctors to take me seriously. It traumatized me.

User 34

In another video, a user made the following announcement:

I was diagnosed with lupus today after 10 years and 14 different sexist doctors, they finally found out I wasn’t just “overtired and dehydrated.”

User 20

Health care workers attributing users’ lupus symptoms to mental health causes seemed to be a common experience among those who experienced medical gaslighting (5/8, 63%). A TikTok video started with the following words:

Been sick since 2010. No doctor would listen.

User 2

The user then showed stock photos of 3 physicians, each depicted as saying, “It’s just anxiety, it’s just anxiety, it’s just anxiety.” The user finished the video by rolling their eyes at the camera and displaying the following words:

Finally diagnosed with lupus, rheumatoid arthritis, among other things, after over a decade...it became medical negligence a LONG time ago. They seriously need to stop telling people that.

User 2

Notably, in 4 (18%) of the 22 videos, the users’ negative experiences with health care workers directly caused them to pursue naturopathic practitioners to treat their lupus.

**Common hashtags explained.**

**Hashtag and description**

- #medicalgaslighting
  - Describes medical providers minimizing symptoms or incorrectly attributing symptoms to a behavioral or psychological cause
  - Primarily developed to describe the experiences of women and Black, Indigenous, and patients from racial and ethnic minority groups [18,19]
  - Popularized in recent years by publications such as *The New York Times* and *The Atlantic* [18,19]
- #spoonie
  - An identity for an individual who experiences limited energy, often due to a chronic illness
  - Derived from “The Spoon Theory,” written by Miserandino [20], a blogger with lupus; the theory describes spoons as units of energy that everyone starts their day with; however, people with a chronic illness only get a few spoons at the start of the day, whereas others get an excess of spoons [21]
- #chemotherapy
  - Used to describe immunosuppressive treatments for lupus, such as methotrexate
  - Possibly popularized by Selena Gomez, a singer and actor who has systemic lupus erythematosus; in a 2015 interview with *Billboard*, she shared that she received chemotherapy to treat her lupus [22,23]; this was met with backlash from people who thought that the term “chemotherapy” should be reserved for patients with cancer [23]
- #invisibleillness
  - An illness with symptoms that are predominantly “invisible” to others; this may lead to misunderstandings from others and diagnostic difficulties [24,25]

### Theme 3: TikTok Users Tended to Portray Nonpharmacological Interventions, Such as Diet and Naturopathic Remedies, Positively, Whereas Pharmacological Treatments Were More Commonly Portrayed Negatively or Referred to as “Chemotherapy”

Of the 153 videos, 58 (37.9%) involved experiences with LE treatment, of which 41% (n=24) were on pharmacological treatments, and 28% (n=16) were on nonpharmacological treatments. Overall, 22% (13/58) involved positive experiences with treatment, and 36% (21/58) involved negative experiences with treatment.

Nonpharmacological treatments tended to be portrayed positively; 9 (56%) of the 16 videos that mentioned nonpharmacological treatments depicted a good experience. The majority of positive experiences with treatment involved nonpharmacological treatments (8/13, 62%), primarily diet (4/8, 50%) and naturopathic remedies (4/8, 50%). Furthermore, nonpharmacological treatments were often credited for disease remission; of the 9 videos that attributed LE remission to treatment, 6 (67%) cited nonpharmacological treatments, whereas only 3 (33%) cited pharmacological treatments. A user stated as follows:

Natural medicine saved my life...[I was] told that I would be on pharmaceuticals for life and that I would never be able to exercise again, I could barely walk, I wouldn’t be able to have kids, I wouldn’t be able to have a job...But luckily, I didn’t listen. Because if I did, I don’t know where I would be today. Instead, I run three different businesses, I found movement that works for me, I’ve completely reversed all fertility issues, I’m in remission from lupus, and most of my markers are completely normal. The secret to my healing, you may be asking? Well, it’s the food that you eat, the herbs that you put into your body, and the habits that you practice on a daily basis that set the course for your entire life.

User 26

By contrast, pharmacological treatments tended to be portrayed negatively; of the 24 videos in which they were mentioned, they were depicted negatively in 10 (42%) and positively in only 3 (13%). Approximately three-fourths (16/21, 76%) of the videos about negative experiences with treatment involved pharmacologic treatments. Negative experiences included side effects (6/16, 38%); injection pain (2/16, 13%); and distress or difficulty with medication management (5/16, 31%) such as remembering to take pills, feeling as though they had too many prescriptions, having an emotional reaction to taking a medication, and relying on perishable and expensive prescriptions.

Furthermore, immunosuppression was repeatedly referred to negatively with the term “chemotherapy.” In total, the term was used by 5 (7%) of the 76 users in 16 (10.5%) of the 153 videos and seemed to be used to portray the severity of disease or the gravity of treatment measures. A user relayed their experience with a flare:

I found out 3 years ago I had lupus. I had to have chemo. It’s been manageable, but last week I had a bad flare up and ended up in the hospital to find out it’s damaged my kidneys, and the doctors are talking about chemo again.

User 23

Another user stated as follows:

I am so physically exhausted from this disease and chemo but this is your reminder—don’t give up.

User 34

### Theme 4: LE Symptoms, Particularly Musculoskeletal Symptoms and Fatigue, Interfered With Users’ Daily Functioning

Of the 153 videos, 29 (19%) referenced LE symptoms or treatments interfering with basic, instrumental, social, educational, or occupational functioning. Musculoskeletal symptoms and fatigue were the most common symptoms to directly overlap with codes related to interference with functioning.

Musculoskeletal symptoms tended to interfere with basic activities of daily living the most; 15 (83%) of the 18 instances of interference with basic functioning were directly attributable. Musculoskeletal symptoms, such as joint pain and stiffness, primarily affected ambulation (8/15, 53%). A user stated as follows:

A lot of days, my body hurts so badly that I don’t possibly know how to get out of bed or survive for the next few hours.

User 70

Another user showed footage of themselves struggling to perform a variety of activities such as sit on a toilet, grip a marker, open a bottle of juice, and stand from a seated position. During this footage, the user displayed the following subtitles:

What it’s like living with lupus. My joints get stiff. Doing normal things are a struggle now. Lupus affects my hands, wrists, and knees. I was just diagnosed and I hope to see improvement soon.

User 48

By contrast, fatigue resulting from LE seemed to be more likely to interfere with social, occupational, and educational functioning, contributing to 5 (56%) of the 9 references within these categories. A user talked about how fatigue affected their schooling:

You’re worried about going back to school because you literally can’t do anything without 15 hours of sleep, and you can’t get your schoolwork done, and you can’t study enough, and it’s horrible.

User 10

Another user talked about feeling fatigued after driving to see a friend:

I had lunch with a friend today. I drove there and drove home, so naturally I am fatigued now. Good, bad, ugly, that a single activity can put me on the couch. I am not worried, but this is a reality.

User 11

Interestingly, 4 (5%) of the 76 users included the word “spoonie” in their TikTok videos, a term that has become an identity for individuals who experience fatigue from chronic illnesses.

### Theme 5: Although TikTok Users Frequently Had Strong Support Systems, Feelings of Isolation or Misunderstanding Were Often Attributed to Battling an “Invisible Illness”

Of the 153 videos, 16 (10.5%) depicted users’ support systems, which were composed of partners (n=8, 50%), family members (n=5, 31%), other TikTok users (n=3, 19%), and friends (n-3, 19%). TikTok users frequently expressed gratitude for the assistance they received from support people in navigating their LE symptoms and treatment. A user showed footage of their hospitalization for LE and displayed the following words:

It’s been a rough few weeks. I couldn’t express how grateful I am for my support system. My family. I couldn’t have done it without you guys.

User 73

Another user, who similarly filmed their TikTok video when they were hospitalized for LE, wrote as follows:

I’m not recovering at the rate I hoped I would by now. I keep watching time go by as the pain gets worse as I lay here...You’re left with the emptiness and questions what you did to deserve this and why you’re here, and the only escape you have is those short few minutes you get a call from a friend or family member and can pretend it isn’t happening.

User 72

However, TikTok users also reported discouraging interactions with others (14/153, 9.2%), including with people from school or work (n=3, 21%), friends (n=3, 21%), family (n=2, 14%), service industry workers (n=2, 14%), partners (n=1, 7%), other TikTok users (n=1, 7%), and neighbors (n=1, 7%). Most of these interactions arose from misunderstandings of LE. Users felt that because LE is primarily an “invisible illness,” with many of its signs and symptoms not visibly apparent to others, others did not always recognize their needs; for example, a user’s video provided a list of “things people with lupus are tired of hearing,” which included comments such as “You can’t be tired, you haven’t done anything all day,” “You don’t look sick,” “You’re using it as an excuse to be lazy,” and “It’s not that bad” (User 41).

These misunderstandings led to feelings of isolation. A user, who filmed themselves lying in bed, commented as follows:

[Lupus is] so isolating because no one understands what you’re going through. I just feel lame having to leave a group setting to have a flare up until your body goes back to normal after a few hours.

User 44

In all, of the 14 videos, 10 (71%) referred to LE as an “invisible illness” through hashtags, captions, or direct quotes, and 6 (43%) that were coded with “negative experiences with others” were also coded with “invisible illness.”

## Discussion

### Principal Findings

This study represents the first qualitative and content analysis of TikTok videos involving personal experiences of users with LE. Patients are increasingly using social media to learn and share information about their health conditions [9-11,26]. Thus, social media provides a crucial fund of patient experiences that can be used to extract clinically relevant patient-centered information for clinicians that may ultimately improve patient care.

In this study, we found that TikTok videos on LE experiences have extensive audiences, garnering millions of views and high user engagement. Videos that mentioned mucocutaneous symptoms of LE, such as hair loss and rash, were pervasive among this sample, with *mucocutaneous symptoms* being the second most frequent code used. Consistent with findings of previous qualitative and survey studies, mucocutaneous symptoms seemed to be major drivers of poor mental health and negative body image among TikTok users with LE [27-33]. Our study suggests the need to regularly assess for mental health and body image concerns in patients with LE, especially among those with active dermatologic symptoms. It also underscores the importance of treating hair loss and rash to mitigate mental health burdens in this population (Textbox 3).

**Clinical applications of themes.**

**Themes and clinical applications**

- Mucocutaneous symptoms had profound effects on the mental health and body image of TikTok users with lupus erythematosus (LE).
  - Treatment of hair loss and rash is important for quality of life and mental health of patients with LE.
  - Mental health should be regularly assessed at appointments, particularly for patients with rash or hair loss.
- TikTok users’ negative experiences with health care workers were often derived from diagnostic delays and perceptions of “medical gaslighting.”
  - Clinical strategies such as reflective listening and validation may enhance the clinician-patient relationship and prevent perceptions of medical gaslighting.
- TikTok users tended to portray nonpharmacologic interventions, such as diet and naturopathic remedies, positively, whereas pharmacologic treatments were more commonly portrayed negatively or referred to as “chemotherapy.”
  - Clinicians should be aware of popular nonpharmacological treatments for LE.
  - Clinicians should engage in informed discussions of the safety and effectiveness of both pharmacological and nonpharmacological treatments with their patients.
- LE symptoms, particularly musculoskeletal symptoms and fatigue, interfered with users’ daily functioning.
  - Treatment should focus on reducing musculoskeletal symptoms and fatigue for patients reporting interference in functioning.
  - Clinicians should assist patients in obtaining mobility devices, disability resources, and occupational and physical therapy that improve daily functioning.
- Although TikTok users frequently had strong support systems, feelings of isolation or misunderstanding were often attributed to battling an “invisible illness.”
  - Involving support people in appointments could be a beneficial way to enhance existing support relationships and educate support people on LE morbidity and disability.

However, we found that pharmacologic therapies might be met with hesitancy by individuals with LE. Pharmacologic treatments were depicted negatively in our sample, with individuals citing side effects such as immunosuppression, weight gain, and fatigue. Notably, the term “chemotherapy” was used in several videos, which portrays the gravity that users associate with receiving immunosuppressive medications. By contrast, nonpharmacologic treatments such as diet and naturopathic remedies were depicted overwhelmingly positively in our sample, a finding that to our knowledge has only been reported once before, in a 2011 qualitative study on attitudes toward medications in South Asian patients with SLE [34]. The uniqueness of this finding could be because users can benefit monetarily from promoting diet or naturopathic remedies through promotion deals on TikTok. However, it is also possible that TikTok users, because they are a nonclinical sample, may have fewer or poorer experiences with clinical medicine and thus prefer nonpharmacologic treatments. Clinicians should be aware of common nonpharmacological options for patients with LE and should be prepared to counsel patients on the safety and effectiveness of these therapies (Textbox 3).

Concordantly, we did find that TikTok users with LE shared primarily negative interactions with the health care system and health care workers. Many of these experiences centered on instances of “medical gaslighting,” which users felt resulted in diagnostic delays. Diagnostic delays are well documented in SLE qualitative and quantitative research and can have significant mental health ramifications for patients [3,35-39]. However, only a few qualitative studies have explored patients’ perceptions of diagnostic delays resulting from physicians downplaying LE symptoms, with only 1 prior study capturing the term “gaslighting” in its analysis [3,38,39]. To maintain the therapeutic relationship, clinicians should combat perceptions of medical gaslighting through strategies such as validation and reflective listening (Textbox 3) [40].

Our sample had high symptom burden and frequently described how musculoskeletal symptoms and fatigue were interfering with daily functioning. TikTok users did not always feel that others understood these symptoms, leading to feelings of isolation and the thought that they have an “invisible illness.” These ideas have been described in numerous qualitative analyses on LE [3,7,24,31,36,41-43]. Even so, overall, TikTok users demonstrated robust social support systems made up of friends, family, and partners. This is important because social support has been associated with improved mental health in patients with SLE, whereas a lack of substantial social support has been associated with increased disease activity [24,44-47]. These combined findings suggest the importance of educating patients’ support people on LE morbidity and disability to facilitate successful support relationships (Textbox 3).

### Strengths

This study has several notable strengths. First, because our qualitative data were derived from a nonclinical sample, we may have captured voices from individuals with diverse experiences with the health care system [8]. Second, because patient experiences were collected without interaction with the study team, patient experiences were unbiased by researcher presence or preset interview questions [48,49]. Third and last, in contrast to existing qualitative studies that often have low sample sizes, analyzing TikTok videos allowed us to gather the experiences of 76 distinct users.

### Limitations

Although qualitative studies are inherently not designed to be generalizable because they provide rich, narrative data from the group being studied, it is important to note that this study only examined TikTok content and did not examine content from other web-based platforms [50]. Thus, these findings may not be representative of the entire LE web-based community. Furthermore, as the TikTok videos were sampled from a search revealing the most popular videos with #lupus, our findings may overrepresent ideas in popular videos, while underrepresenting ideas from users with fewer views. Furthermore, in comparison to qualitative studies in which interviewees are promised confidentiality when disclosing their experiences, TikTok videos in our sample were not confidential, and, in fact, were meant to be publicly shared. This means that patient experiences were subject to social desirability bias, and sensitive topics may have been avoided. Finally, demographic data of TikTok users are not publicly available, and thus detailed user demographics could not be characterized in this study.

### Conclusions

TikTok provides a nonclinical, underused platform for qualitative and content analysis of patient experiences. This study summarizes key terminology and content in the LE TikTok community, which may be clinically relevant because a substantial number of patients use social media to obtain medical information [9,10]. Ultimately, this study presents 5 thematic conclusions paired with clinical applications, which offer an enhanced understanding of how the well-being of patients with LE is influenced by symptoms, treatments, support people, and health care experiences.

## Appendix

Multimedia Appendix 1 Final codebook.

## Abbreviations

LE: lupus erythematosus
SLE: systemic lupus erythematosus
