# Supplementary material for: Evaluating the Disease-Related Experiences of TikTok Users With Lupus Erythematosus: Qualitative and Content Analysis
Source: JMIR Infodemiology. 2024 Apr 17;4:e51211. doi: 10.2196/51211 (PMC11063877; doi:10.2196/51211)
Supplement: Multimedia Appendix 1 [file infodemiology_v4i1e51211_app1.docx]

Multimedia Appendix 1. Final Codebook

| Body image |
| --- |
| Description: User shares how lupus symptoms or treatments have affected their appearance and/or confidence about their appearance. |
| Subcategories:   - Positive body image - Negative body image |
| Examples: User shares pictures of their hair loss. User feels more confident about their appearance after getting treatment for their lupus. User shows before and after photographs of their rash. |
| Exclusions: User is distressed about lupus symptoms such as joint pain or fatigue, but does not relate this to their appearance, body image, or confidence about their appearance. |

| Coexisting medical conditions |
| --- |
| Description: User suggests that they have an acute or chronic medical or psychiatric condition other than lupus. |
| Examples: User states that they have bipolar disorder. |
| Exclusions: User refers to feeling temporarily anxious, without suggesting having a diagnosis of an anxiety disorder. |

| Complementary and Alternative Medicine principles |
| --- |
| Description: Personal opinions regarding complementary and alternative medicine principles or providers. |
| Examples: User states that they believe in alternative medicine practices for treating lupus. |
| Exclusions: User discusses their own experience with a specific complementary and alternative treatment (this is coded under “treatments”). |

| Coping mechanisms |
| --- |
| Description: User refers to how they relax, engage in self-care, or what coping mechanisms they use to manage distress resulting from their lupus. |
| Examples: User refers to coping mechanisms such as hot baths, psychotherapy, candles, support groups, or religious/spiritual beliefs. |
| Exclusions: User refers to treatments of lupus (this is coded under “treatments”). |

| COVID-19 |
| --- |
| Description: User refers to COVID-19. |
| Examples: User discusses the effect that the COVID-19 pandemic has had on their experience with lupus. |

| Flare |
| --- |
| Description: User refers to personal experiences with flares, significant disease exacerbation, or triggers of flares. |
| Examples: User refers to the sun being a trigger for flares of their lupus. User states that they are currently in a disease flare. |

| Health care experience |
| --- |
| Description: User discusses or depicts personal health care experiences related to their lupus. |
| Subcategories:   - Positive health care experience - Negative health care experience - Positive experience with a health care worker - Negative experience with a health care worker - Diagnostic delay - Experiences with diagnostic testing |
| Examples: User describes their experience getting their blood drawn. User describes their experience with a health care worker, such as a physician or nurse. |

| Hospitalizations |
| --- |
| Description: User refers to a personal experience of being hospitalized for lupus. |
| Examples: User states that they just got out of the hospital, user shows pictures of being in the hospital. |
| Exclusions: User refers to an outpatient appointment (rather than inpatient), user is hospitalized but for a non-lupus related reason. |

| Humor |
| --- |
| Description: User utilizes humor in their video. |
| Examples: User makes light of their lupus experience, user employs dark humor, user uses music, memes, audio, or other effects to make their content humorous. |

| Interference with functioning |
| --- |
| Description: User refers to lupus or lupus treatments affecting their functioning. |
| Subcategories:   - Interference with basic activities of daily living (ambulating, feeding, dressing, personal hygiene, continence, toileting)^1^ - Interference with instrumental activities of daily living (transportation and shopping, managing finances, shopping and meal preparation, housecleaning and home maintenance, managing communication with others, managing medications)^1^ - Interference with work or school - Interference with socialization |
| Examples: User misses work, school, or a social event due to lupus symptoms. |

| Invisible illness |
| --- |
| Description: User refers to the “invisible” nature of lupus; more specifically, others may not know that they have lupus because it is not visibly apparent, or others may not know the extent to which their lupus affects them. |
| Examples: User states that no one knows how much pain they are in because they look fine on the outside. |
| Exclusions: User discusses how a health care worker did not believe that they had lupus (this would be coded under “health care experience”). |

| Mental health |
| --- |
| Description: User refers to how lupus affects their emotional and mental wellbeing. |
| Examples: User is overwhelmed about their lupus diagnosis. User is distressed about their hair loss. |
| Exclusions: Mood disorders or psychiatric diagnoses (this is coded under “coexisting medical conditions”). |

| Outreach and advocacy |
| --- |
| Description: User expresses outreach and/or advocacy principles, including user spreading awareness about lupus, promoting lupus-related organizations, and/or discussing lupus and its relationship with social justice, current events, or public health. |
| Subcategories:   - Health advice: User gives health advice to other users who may have lupus. - Education: User educates other users about lupus. |
| Examples: User wants to raise money for a lupus non-profit organization, user educates their viewers about common signs and symptoms of lupus, user discusses health care disparities in lupus care. |

| Parenthood |
| --- |
| Description: User mentions how lupus affects their experience of being a parent, or simply refers to being a parent. |
| Examples: User states that they do not have the energy to play with their child. |

| Prognosis |
| --- |
| Description: User refers to the longitudinal or future course of lupus. |
| Subcategories:   - Chronicity of lupus - Morbidity of lupus - Mortality of lupus |
| Examples: User mentions that lupus does not have a cure. User mentions that people can die from lupus. User mentions the long-term consequences of lupus on quality of life. |
| Exclusions: Distress about current symptoms without reference to the future. |

| Remission and low disease activity |
| --- |
| Description: User describes personal experiences with remission from lupus or improvement in signs/symptoms of lupus. |
| Examples: User states that their hair is growing back. User states that their energy is improving. |

| Reproductive health |
| --- |
| Description: User mentions their feelings or experiences regarding lupus and fertility, pregnancy, and/or miscarriages. |
| Examples: User worries about not being able to have children due to lupus. |

| Romance and sexuality |
| --- |
| Description: User refers to personal experiences with dating, romance, and/or sexuality while having lupus. |
| Examples: User mentions going on a date while having lupus. |

| Self-expression |
| --- |
| Description: User uses self-expression, including art or dance, to relay their experience with lupus. |
| Examples: User shows a drawing that they feel represents lupus. |

| Signs |
| --- |
| Description: User refers to their personal diagnostic, imaging, or physical exam results relating to lupus. |
| Subcategories:   - ANA |
| Examples: User mentions that they had a positive ANA when they were diagnosed. |

| Support animals |
| --- |
| Description: User refers to getting support, including emotional or functional support, from an animal. |
| Examples: User states that their dog makes them feel better when their lupus is flaring. |
| Exclusions: User mentions an animal, or shows images and/or video of an animal, with no reference to that animal being a support. |

| Support of others |
| --- |
| Description: User describes their experiences with other people, whether positive or negative, in the context of their experience with lupus. |
| Subcategories:   - Positive experiences with others: Other people contributing positively to the user’s experience with lupus. - Negative experiences with others: Other people contributing negatively to the user’s experience with lupus.   - Ableism: Relating to negative experiences with disability accommodations, stigma around disability, and other forms of ableism. |
| Examples: User states that their friend has helped them go to appointments, user states that their school has not provided disability accommodations, user states that someone said something that was hurtful or unhelpful relating to their lupus. |
| Exclusions: Experiences with health care workers (this would be coded under “health care experience”). |

| Symptoms |
| --- |
| Description: User refers to their personal lupus symptom(s). |
| Subcategories:   - Cardiovascular   - Vascular:     - Raynaud’s     - Vasculitis - Constitutional   - Weight loss   - Weight gain   - Malaise   - Night sweats   - Fatigue - Ears, Nose, Throat - Endocrine - Eyes - Gastrointestinal - Genitourinary - Hematologic/Lymphatic - Mucocutaneous   - Rash   - Itch   - Hair   - Ulcers   - Sicca   - Photosensitivity - Musculoskeletal - Neuropsychiatric - Renal - Respiratory |
| Examples: User states that they have joint pain (“musculoskeletal”) and are too tired to get out of bed (“fatigue”). |

| Treatment |
| --- |
| Description: User refers to a personal experience with, or their opinion regarding, a treatment for lupus. |
| Subcategories:   - Positive experience with treatment - Negative experience with treatment   - Side effects - Transplant/Dialysis - Pharmacologic treatments   - Immunosuppressants/Immunomodulatory drugs   - Topicals - Non-pharmacologic treatments:   - Complementary and alternative medicine treatments   - Diet   - Topicals   - Photoprotection - Uses the term “chemotherapy” |
| Examples: User discusses their experience with administering belimumab. |

References:

1. Edemekong PF, Bomgaars DL, Sukumaran S, et al. Activities of Daily Living. Treasure Island (FL): StatPearls Publishing, 2022.
